# Knowledge, Attitudes, and Practices Related to Diabetes Mellitus Among Hospital Outpatients and Their Companions in Herat, Afghanistan: A Cross‐Sectional Study

**DOI:** 10.1002/puh2.70256

**Published:** 2026-04-30

**Authors:** Mohammad Masudi, Mohammad Shafi Saljuqi, Fatemah Rezaei, Zhila Arjmand, Parwin Arsin, Ali Rahimi, Enayatollah Ejaz, Fahim Ahmadi, Nasar Ahmad Shayan

**Affiliations:** ^1^ Department of Curative Medicine Faculty of Medicine Jami University Herat Afghanistan; ^2^ Department of Pediatrics Faculty of Medicine Herat University Herat Afghanistan; ^3^ Department of Curative Medicine Faculty of Medicine Herat University Herat Afghanistan; ^4^ Department of Epidemiology and Biostatistics Schulich School of Medicine and Dentistry Western University London Ontario Canada

**Keywords:** Afghanistan, attitude, diabetes mellitus, knowledge, outpatients, practice, public health

## Abstract

**Objectives:**

This study investigates the knowledge, attitudes, and practices (KAP) related to diabetes mellitus (DM) among outpatients in Herat, aiming to identify key gaps and inform educational and policy interventions.

**Methods:**

An outpatient‐based cross‐sectional study, using a structured‐, validated‐, and interviewer‐administered questionnaire, was conducted among 421 adult outpatients at Herat Regional and Jami Hospitals. Descriptive statistics, chi‐square tests, and logistic regression were performed in SPSS 27 to explore associations between KAP levels and sociodemographic factors.

**Results:**

Overall, 54.4% had good knowledge, 51.3% positive attitudes, and 52.5% good practices. Knowledge correlated with education, diabetes education, and family history (*p* < 0.001). Positive attitudes were more common in singles, urban residents, and media users (*p* < 0.05). Good practices were higher in females, housewives, and those working fewer hours (*p* < 0.05). In multivariable analysis, knowledge was associated with age 25–34 (AOR 3.94, *p* = 0.044), rural residence (AOR 0.20, *p* = 0.032), university education (AOR 0.19, *p* = 0.028), normal BMI (AOR 0.11, *p* = 0.019), and family history of Type 2 diabetes (AOR 0.25, *p* = 0.007). Positive attitudes were linked to working 2–9 h (AOR 3.41, *p* = 0.012), whereas medium income decreased odds (AOR 0.23, *p* = 0.013). Good practices were associated with female sex (AOR 0.13, *p* = 0.010), being married (AOR 0.20, *p* = 0.047), working 2–9 h (AOR 3.50, *p* = 0.033), literacy without schooling (AOR 12.31, *p* = 0.012), and prior diabetes education (AOR 8.90, *p* < 0.001). High income showed a negative association (AOR 0.21, *p* = 0.040).

**Conclusion:**

Although moderate KAP levels were observed, critical gaps remain, particularly in lifestyle practices and routine monitoring. Comprehensive public health strategies including structured education, media outreach, and culturally tailored interventions are vital to improve diabetes awareness and management in Afghanistan.

## Graphical abstract

Moderate knowledge, attitudes, and practices (KAP) toward diabetes mellitus were observed among outpatients and their companions in Herat, with significant sociodemographic disparities. Targeted health education, media engagement, and culturally tailored interventions are needed to address gaps in lifestyle practices and routine monitoring.

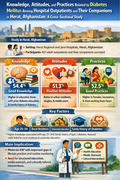

## 1 Introduction

Diabetes mellitus (DM) is a chronic metabolic disorder characterized by persistent hyperglycemia, which over time can lead to complications affecting the cardiovascular system, kidneys, eyes, and peripheral nerves [1]. It is recognized as a major contributor to global morbidity and mortality, currently affecting an estimated 830 million individuals worldwide, with the majority living in low‐ and middle‐income countries [2]. Despite its high prevalence, a substantial proportion of individuals remain undiagnosed or inadequately treated [2]. In 2021, diabetes was responsible for approximately 1.6 million deaths globally, and projections indicate that diabetes‐related deaths could reach 592 million by 2035 [2, 3]. The economic burden is equally significant, with global healthcare expenditures related to diabetes estimated at $966 billion in 2021 [4].

Regionally, neighboring countries face a high burden of diabetes as well. In Iran, the prevalence among adults aged 25 years and older was estimated at 10.6% in 2016, affecting over 5.1 million individuals [5]. Pakistan, with approximately 33 million adults living with diabetes as of 2021, ranks among the countries with the highest prevalence globally [6].

In Afghanistan, the prevalence of diabetes among adults aged 20–79 years is estimated at approximately 10.9%, translating to around 1 million diagnosed cases, with an additional 1–2 million potentially undiagnosed [7]. In 2020 alone, diabetes contributed to 5750 deaths in the country, accounting for about 2.48% of all recorded deaths [8]. These figures underscore the growing public health significance of diabetes in Afghanistan, where access to structured screening and long‐term care remains limited.

DM is strongly associated with numerous long‐term complications that contribute substantially to morbidity and mortality [9]. These include microvascular complications, such as retinopathy and nephropathy, and macrovascular complications, including cardiovascular diseases. Approximately 32.2% of individuals with Type 2 DM (T2DM) worldwide are affected by cardiovascular disease, which is the leading cause of death (10) in this population [10]. Moreover, retinopathy affects nearly all patients with Type 1 diabetes and more than 60% of those with Type 2 diabetes within two decades of diagnosis [11]. Diabetes is also responsible for over 1 million amputations globally each year and exacerbates outcomes in comorbid conditions such as tuberculosis, where patients with diabetes face higher mortality rates (10.3%) compared to people without diabetes (7.6%) [3, 12]. These complications significantly impact patients’ quality of life and impose substantial healthcare costs [13].

Understanding diabetes is crucial for its early detection and effective management [14]. Numerous studies indicate that patients with better knowledge and awareness of their condition exhibit more positive attitudes and healthier practices, seek medical care earlier, and achieve better health outcomes [15]. Conversely, insufficient knowledge and misconceptions about the disease often result in poor disease control, delayed diagnosis, and increased risk of complications [16]. Therefore, assessing patients’ knowledge, attitudes, and practices (KAP) is essential. KAP not only influences individual disease management but also informs healthcare policies and programs [17]. In resource‐limited settings such as Afghanistan, KAP studies are particularly valuable, as they help bridge gaps in health system data and guide evidence‐based interventions [18].

In Afghanistan, few studies have examined the prevalence and risk factors of diabetes. A population‐based survey in Herat reported a prevalence of 9.9%, with only 3.3% of cases previously diagnosed, highlighting substantial underdiagnosis and low public awareness [19]. Another study found a similar prevalence (9.7%) and identified significant associations with male gender, physical inactivity, increased waist circumference, and a positive family history [20]. However, to date, no comprehensive study has been conducted in Afghanistan assessing KAP related to DM among Herat populations.

Therefore, this study aimed to assess the KAP related to DM among hospital outpatients in Herat, Afghanistan, in order to identify awareness gaps and inform targeted educational and public health interventions.

## 2 Methods

### 2.1 Study Setting and Design

A facility‐based cross‐sectional study was conducted between January 1 and December 25, 2024, in Herat City, Afghanistan. Herat was selected because it is one of the largest urban centers in the country, with an estimated population of **781,380 in 2026**, and serves as a major healthcare hub for western Afghanistan. The study was conducted in the general outpatient departments of **Herat Regional Hospital** and **Jami Hospital**. Herat Regional Hospital is the largest public referral hospital in the region and provides services to approximately **1500–2000 patients per day**, reflecting its broad catchment population and high patient turnover. Inclusion of these hospitals enabled recruitment from a diverse outpatient population in an urban setting.

### 2.2 Sample Size and Sampling Method

Sample size was determined using Epi Info software, assuming a 95% confidence level, 50% expected prevalence, 5% margin of error, and a design effect of 1.0. This yielded a required minimum of 385 participants. To enhance statistical precision and account for possible nonresponses, the sample was deliberately oversampled, resulting in 421 completed questionnaires. Participants were selected consecutively during regular clinic hours across the study period using a non‐probability convenience sampling approach. Although efficient, this method introduces potential selection bias, which is acknowledged as a limitation of the study.

### 2.3 Inclusion and Exclusion Criteria

Eligible participants were adults aged 18 years and older who were either patients attending the general outpatient departments for any reason or adult companions accompanying them during the study period. The sample was not restricted to diabetes outpatient clinics and did not require participants to have a prior diagnosis of DM. Individuals younger than 18 years and those who were critically ill, cognitively impaired, unable to communicate effectively, or unable to provide informed consent were excluded from participation.

### 2.4 Data Collection Instrument

A structured, interviewer‐administered questionnaire was used to collect data. The tool was adapted from validated KAP studies [21, 22, 23] and was contextualized for diabetes awareness in the Afghan setting. The questionnaire was translated into Persian using forward‐ and back‐translation techniques. Content validity was reviewed by 10 research experts, and a pilot study involving 30 participants confirmed reliability, yielding Cronbach's alpha scores above 0.810 across all domains. Data were collected through face‐to‐face interviews conducted in a private space within the hospital, with responses recorded on paper forms.

### 2.5 Variables and Measurement

#### 2.5.1 Sociodemographic Variables

Information collected included age, sex, marital status, education level, occupation, daily working hours, ethnicity, income type, economic status, family size, BMI (self‐reported height and weight), place of residence (urban/rural), access to mass media, smoking and alcohol use, family history of diabetes (Type 1 or 2), prior diabetes education, and main source of diabetes information.

#### 2.5.2 Knowledge Assessment

Knowledge of DM was assessed using 29 close‐ended questions addressing general understanding, symptoms, complications, and prevention. Correct answers were scored as 1, incorrect or unsure responses as 0, yielding a total score range of 0–29. On the basis of the median score, participants were categorized as having “poor” or “good” knowledge.

Example questions included:

- “Diabetes is a condition characterized by increased blood glucose.”
- “Obesity and unhealthy diet are risk factors for diabetes.”
- “Diabetes can lead to kidney failure and limb amputation.”

#### 2.5.3 Attitude Assessment

Attitudes were measured through 11 statements rated on a five‐point Likert scale (1 = strongly disagree to 5 = strongly agree). Final scores ranged from 11 to 55 and were categorized using the median split.

Example attitude statements included:

- “I believe that regular screening for diabetes is important.”
- “Family and social support are essential in managing diabetes.”
- “Diabetes seriously affects quality of life and marital relationships.”

#### 2.5.4 Practice Assessment

Practice‐related behavior was assessed with nine items using a five‐point Likert scale (1 = not at all to 5 = very frequent). Unhealthy behaviors were reverse‐scored. Final scores ranged from 9 to 45 and were categorized using the median split.

Example practice questions included:

- “Do you check your blood sugar regularly?”
- “Do you avoid consuming sugary foods such as cake and soda?”
- “If you or a family member had diabetes, would you seek care from a doctor?”

### 2.6 Instrument Reliability

Cronbach's alpha coefficients were as follows: knowledge = 0.812, attitude = 0.793, and practice = 0.821—demonstrating good internal consistency across all domains.

## 3 Results

The study population consisted of a diverse demographic, with the majority aged ≥35 years (34.2%) and predominantly female (66.0%). Most participants were currently married (59.1%) and belonged to the Tajik ethnic group (46.8%). Regarding occupation, the highest proportion included those classified as housewives (36.8%), whereas 43.2% worked 7–9 h daily. Educationally, university graduates comprised the largest group (38.7%). A significant proportion resided in urban areas (80.5%) and had a medium economic status (43.2%), with income variability observed in 62.2%. Normal weight was the most prevalent BMI category (53.7%). Access to mass media was high (94.5%), and whereas 93.3% had heard about diabetes, only 26.6% had received formal education on it. A family history of diabetes was reported by 34.6%, with Type 2 being the most common (58.4%). Among those aware of diabetes, healthcare workers were the primary source of information (43.2%) (Table 1).

**TABLE 1:** This table shows the sociodemographic characteristics of participants.

Variables	*N* (%)
Age
18–24	138 (32.8)
25–34	139 (33.0)
≥35	144 (34.2)
Sex
Male	143 (34.0)
Female	278 (66.0)
Marital status
Single/Previously married	172 (40.9)
Currently married	249 (59.1)
Occupation
Employee/Unskilled worker	110 (26.1)
Housewife	155 (36.8)
Other (farmer, student, other)	156 (37.1)
Working hours
1–6 h	112 (26.6)
7–9 h	182 (43.2)
10–15 h	127 (30.2)
Education
Cannot read and write	102 (24.2)
Can read and write	47 (11.2)
School	109 (25.9)
University	163 (38.7)
Ethnicity
Pashtun	125 (29.7)
Tajik	197 (46.8)
Others (Hazara, Uzbek, Turk)	99 (23.5)
Economic status
High	126 (29.9)
Medium	182 (43.2)
Low	113 (26.8)
Types of income
Constant	159 (37.8)
Variable	262 (62.2)
Family member
≤5	136 (32.3)
5–10	245 (58.2)
≥11	40 (9.5)
Living place
Urban	339 (80.5)
Rural	82 (19.5)
BMI category
Underweight	47 (11.2)
Normal weight	226 (53.7)
Overweight/Obese	148 (35.2)
Access to mass media?
Yes	398 (94.5)
No	23 (5.5)
Have you heard about diabetes?
Yes	393 (93.3)
No	28 (6.7)
Have you ever had education about diabetes?
Yes	112 (26.6)
No	309 (73.4)
Is there family history of diabetes in your family?
Yes	146 (34.6)
No	275 (65.3)
If there is, then which type of diabetes?
Type 1	62 (41.6)
Type 2	87 (58.4)
If you heard about diabetes, then what is your source?
Media	82 (19.5)
HWCs	182 (43.2)
From friends and family and others	157 (37.3)

The majority of participants demonstrated a good understanding of diabetes, with 86.9% correctly identifying it as a state of increased blood glucose and 84.6% recognizing high blood sugar as a key sign of the disease. Awareness of major risk factors was also high, with 78.1% acknowledging obesity and being overweight as contributors, 80.0% associating unhealthy food and a sedentary lifestyle with the condition, and 73.6% identifying insufficient exercise as a risk factor. Regarding symptoms, excessive thirst (86.0%) and increased urine frequency (77.0%) were the most recognized, followed by excessive hunger (65.6%) and weight loss (59.6%). A substantial proportion of respondents (80.5%) were aware that slow wound healing is a sign of diabetes, and 76.2% knew that insulin injections are available for management, whereas 84.8% acknowledged the availability of specific tablets and pills. The role of lifestyle modifications in diabetes control was well understood, with 82.9% recognizing the importance of regular exercise and healthy eating, and 66.5% acknowledging weight loss as a management strategy. Additionally, 76.7% were aware that diabetes could lead to eye problems, including blindness, whereas 72.9% recognized its link to heart failure, and 78.1% understood its association with limb amputation (Table 2).

**TABLE 2:** Knowledge toward diabetes (diabetes mellitus [DM]).

Question	Yes (*N*, %)	No (*N*, %)	Not sure (*N*, %)
Diabetes is caused by decreased insulin production?	228 (54.2)	101 (24.0)	92 (21.9)
Diabetes is caused by reduced body response to insulin?	185 (43.9)	123 (29.2)	113 (26.8)
Diabetes is a state of increased blood glucose?	366 (86.9)	24 (5.7)	31 (7.4)
Is diabetes a curable disease?	260 (61.8)	105 (24.9)	56 (13.3)
Can diabetes harm any body organ?	310 (73.6)	50 (11.9)	61 (14.5)
Increased age is a risk factor for diabetes	307 (72.9)	62 (14.7)	52 (12.4)
Genetic or family history of diabetes is a risk factor for diabetes	314 (74.6)	40 (9.5)	67 (15.9)
Obesity and being overweight are risk factors for diabetes	329 (78.1)	43 (10.2)	49 (11.6)
Pregnancy is a risk factor for diabetes	238 (56.5)	66 (15.7)	117 (27.8)
Unhealthy food and a sedentary life are risk factors for diabetes	337 (80.0)	38 (9.0)	46 (10.9)
Insufficient exercise is a risk factor for diabetes	310 (73.6)	51 (12.1)	60 (14.3)
Increased urine frequency is a symptom of diabetes	324 (77.0)	36 (8.6)	61 (14.5)
Excessive thirst is a symptom of diabetes	362 (86.0)	24 (5.7)	35 (8.3)
Excessive hunger is a symptom of diabetes	276 (65.6)	69 (16.4)	76 (18.1)
Weight loss is a symptom of diabetes	251 (59.6)	73 (17.3)	97 (23.0)
High blood sugar is a sign of diabetes	356 (84.6)	22 (5.2)	43 (10.2)
Slow wounds and ulcer repair is a sign of diabetes	339 (80.5)	32 (7.6)	50 (11.9)
Insulin injections are available for diabetes management	321 (76.2)	51 (12.1)	49 (11.6)
Specific tablets, pills, and capsules are available for diabetes management	357 (84.8)	31 (7.4)	33 (7.8)
Regular exercise and healthy foods help in controlling diabetes	349 (82.9)	27 (6.4)	45 (10.7)
Eye examination is important in controlling diabetes	272 (64.6)	62 (14.7)	87 (20.7)
Fingers and toes examination are important for diabetes control	255 (60.6)	77 (18.3)	89 (21.1)
Losing weight is a way for diabetes management	280 (66.5	53 (12.6)	88 (20.9)
Diabetes causes eye problems, even eye blindness	323 (76.7)	43 (10.2)	55 (13.1)
Diabetes causes heart failure	307 (72.9)	57 (13.5)	57 (13.5)
Diabetes causes kidney failure	217 (51.5)	103 (24.5)	101 (24.0)
Diabetes causes stroke	196 (46.6)	113 (26.8)	112 (26.6)
Diabetes causes limb amputation	329 (78.1)	56 (13.3)	36 (8.6)

The majority of participants demonstrated positive attitudes toward diabetes management and prevention. A significant proportion strongly agreed (63.7%) and agreed (34.2%) that they should be examined for diabetes, whereas 47.5% strongly agreed and 29.9% agreed that family members should also be screened. Support from family and friends was acknowledged as important, with 51.3% strongly agreeing and 37.3% agreeing. Regarding dietary control, 56.3% strongly agreed and 34.4% agreed that avoiding excessive sugar intake is essential. Physical activity as a preventive measure was supported by 44.4% who strongly agreed and 39.7% who agreed. Maintaining a healthy weight in diabetes management was endorsed by 56.1% who strongly agreed and 31.1% who agreed. Additionally, 53.2% strongly agreed and 31.8% agreed that complications can be prevented if blood glucose levels are well controlled. In terms of smoking cessation, 60.6% strongly agreed and 28.7% agreed about discussing it with their healthcare team. However, perceptions about diabetes’ impact on daily life and marital relationships varied, with 35.4% remaining neutral on its effect on relationships, and 24.0% being neutral about its influence on daily activities (Table 3).

**TABLE 3:** Attitudes and perceptions toward diabetes (diabetes mellitus [DM]).

Question	Strongly agree (*N*, %)	Agree (*N*, %)	Neutral (*N*, %)	Disagree (*N*, %)	Strongly disagree (*N*, %)
I don't mind if others know that I am with DM	183 (43.5)	137 (32.5)	31 (7.4)	52 (12.4)	18 (4.3)
Do you think that you should be examined for DM?	268 (63.7)	144 (34.2)	6 (1.4)	2 (0.5)	1 (0.2)
Do you think family members should be screened for DM?	200 (47.5)	126 (29.9)	87 (20.7)	4 (1.0)	4 (1.0)
Do you think support from family and friends is important in dealing with DM?	216 (51.3)	157 (37.3)	41 (9.7)	4 (1.0)	3 (0.7)
Do you think we should avoid consuming too much sugar for controlling DM?	237 (56.3)	145 (34.4)	31 (7.4)	5 (1.2)	3 (0.7)
DM does not seriously affect the marital relationship	70 (16.6)	81 (19.2)	149 (35.4)	81 (19.2)	40 (9.5)
I don't think DM seriously affects daily activities	64 (15.2)	90 (21.4)	101 (24.0)	117 (27.8)	49 (11.6)
Do you think physical activity can prevent the risk of DM?	187 (44.4)	167 (39.7)	58 (13.8)	6 (1.4)	3 (0.7)
Do you discuss stopping smoking with your healthcare team?	255 (60.6)	121 (28.7)	26 (6.2)	10 (2.4)	9 (2.1)
Do you think maintaining a healthy weight is important in diabetes management?	236 (56.1)	131 (31.1)	44 (10.5)	6 (1.4)	4 (1.0)
DM complications may be prevented if blood glucose level is well controlled	224 (53.2)	134 (31.8)	61 (14.5)	1 (0.2)	1 (0.2)

The majority of participants reported very frequent doctor visits if they or a family member had diabetes (80.8%). In terms of physical activity, 48.0% reported engaging in 30–60 min of daily physical activity very frequently, whereas 34.7% participated in maintaining a healthy weight very frequently. Regarding dietary habits, 37.5% frequently consumed fatty foods, and 34.0% frequently consumed sugary foods like cakes and candy. Carbonated and sweetened beverages were less frequently consumed by 35.9% of participants. Notably, 100.0% reported not at all drinking alcohol, and 79.8% reported not smoking or using tobacco products. Regular blood sugar monitoring was not at all performed by 44.9% of participants (Table 4).

**TABLE 4:** Diabetes‐related health and lifestyle practices.

Question	Very frequent (*N*, %)	Frequent (*N*, %)	Not sure (*N*, %)	Less frequent (*N*, %)	Not at all (*N*, %)
If I or one of my family members has DM, do we go to the doctor?	340 (80.8)	58 (13.8)	10 (2.4)	13 (3.1)	0 (0.0)
Do you consume fatty foods?	52 (12.4)	158 (37.5)	11 (2.6)	166 (39.4)	34 (8.1)
Do you do 30–60 min of physical activity daily? (e.g., brisk walking, house activities, climbing stairs)	202 (48.0)	142 (33.7)	11 (2.6)	49 (11.6)	17 (4.0)
Do you participate in maintaining your healthy weight?	146 (34.7)	108 (25.7)	8 (1.9)	82 (19.5)	77 (18.3)
Do you drink alcohol?	0 (0.0)	0 (0.0)	0 (0.0)	0 (0.0)	421 (100.0)
Do you drink carbonated and sweetened beverages?	69 (16.4)	121 (28.7)	7 (1.7)	151 (35.9)	73 (17.3)
Do you consume more sugary foods like cake and candy?	89 (21.1)	143 (34.0)	9 (2.1)	137 (32.5)	43 (10.2)
Do you smoke or use tobacco products?	17 (4.0)	19 (4.5)	4 (1.0)	45 (10.7)	336 (79.8)
Do you regularly check your blood sugar?	39 (9.3)	69 (16.4)	5 (1.2)	119 (28.3)	189 (44.9)

**FIGURE 1:**
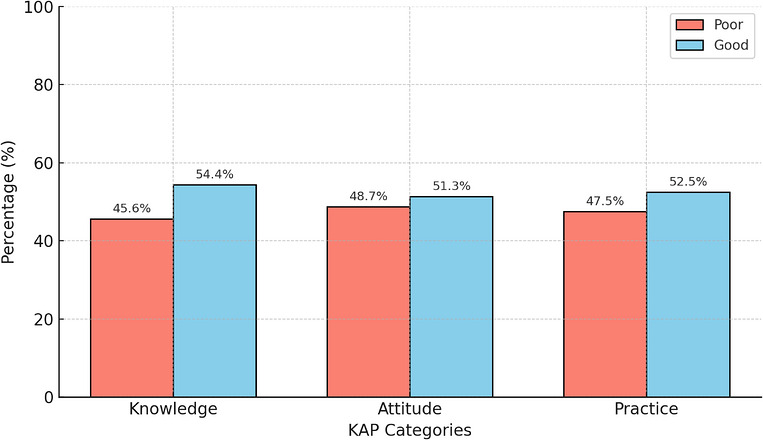
Knowledge, attitude, and practice regarding diabetes mellitus. KAP, knowledge, attitudes, and practices.

In this study, 45.6% of participants had poor knowledge and 54.4% had good knowledge; 48.7% had poor attitude and 51.3% had good attitude, whereas 47.5% showed poor practice and 52.5% demonstrated good practice regarding DM (Figure 1). Females demonstrated higher levels of good knowledge (57.9%) compared to males (47.6%) (*p* = 0.043). Participants with university education had the highest proportion of good knowledge (65.6%) versus those with school‐level education (46.8%), those who could read and write without formal schooling (44.7%), and those who could not read or write (49.0%) (*p* = 0.003). Individuals who had received diabetes education reported significantly higher knowledge (75.9%) compared to those who had not (46.6%) (*p* < 0.001). Those with a family history of diabetes had greater knowledge (68.5%) than those without such history (46.9%) (*p* < 0.001). Among participants with a family history, those with Type 2 diabetes relatives had better knowledge (77.0%) compared to those with Type 1 diabetes relatives (58.1%) (*p* = 0.014). Participants who received information from healthcare workers showed higher knowledge (64.3%) than those who learned from media (53.7%) or from friends, family, and others (43.3%) (*p* = 0.001). Single or previously married participants showed a higher rate of good attitude (57.6%) compared to those currently married (47.0%) (*p* = 0.033). Participants with university education had the highest proportion of good attitude (58.9%), followed by those with school education (55.0%), whereas those who could only read and write (34.0%) and those who could not read or write (43.1%) had the lowest levels (*p* = 0.005). Those living with parents, friends, or alone demonstrated better attitude (56.4%) compared to those living with wife and children (46.6%) (*p* = 0.043). Urban residents showed a significantly more positive attitude (54.3%) than rural residents (39.0%) (*p* = 0.013). Additionally, those with access to mass media had higher attitude scores (52.5%) compared to those without access (30.4%) (*p* = 0.039). Females exhibited significantly higher good practice levels (60.4%) compared to males (37.1%) (*p* < 0.001). Participants working 1–6 h per day had better practice (61.6%) than those working 7–9 h (56.0%) and 10–15 h (39.4%) (*p* = 0.001). Housewives showed the highest good practice (59.4%) compared to employees/unskilled workers (42.7%) and others such as students and farmers (52.6%) (*p* = 0.028). Participants who had heard about diabetes demonstrated better practice (55.3%) than those who had not (46.1%) (*p* = 0.003). Those who had received diabetes education reported higher good practice (55.2%) than those who had not (53.2%) (*p* < 0.001). Participants with a family history of diabetes showed higher good practice (54.4%) than those without such history (48.7%) (*p* < 0.001). Lastly, individuals informed by media had better practice (53.4%) compared to those informed by healthcare workers (52.4%) and by friends or family (47.8%) (*p* = 0.028) (Table 5).

**TABLE 5:** Association of knowledge, attitude, and practice with sociodemographic variables.

	Good knowledge	*p* value	Good attitude	*p* value	Good practice	*p* value
Age categorized
18–24	74 (53.6%)	0.366	72 (52.2%)	0.140	66 (47.8%)	0.154
25–34	82 (59.0%)		79 (56.8%)		82 (59.0%)	
≥35	73 (50.7%)		65 (45.1%)		73 (50.7%)	
Sex
Male	68 (47.6%)	0.043	80 (55.9%)	0.172	53 (37.1%)	<0.001
Female	161 (57.9%)		136 (48.9%)		168 (60.4%)	
Marital status categorized
Single/Previously married	94 (54.7%)	0.930	99 (57.6%)	0.033	85 (49.4%)	0.294
Currently married	135 (54.2%)		117 (47.0%)		136 (54.6%)	
Occupation categorized
Employee/Unskilled worker	54 (49.1%)	0.271	59 (53.6%)	0.057	47 (42.7%)	0.028
Housewife	83 (53.5%)		68 (43.9%)		92 (59.4%)	
Other (farmer, student, other)	92 (59.0%)		89 (57.1%)		82 (52.6%)	
Working hours categorized
1–6 h	66 (58.9%)	0.417	61 (54.5%)	0.408	69 (61.6%)	0.001
7–9 h	99 (54.4%)		96 (52.7%)		102 (56.0%)	
10–15 h	64 (50.4%)		59 (46.5%)		50 (39.4%)	
Education categorized
Cannot read and write	50 (49.0%)	0.003	44 (43.1%)	0.005	47 (46.1%)	0.505
Can read and write	21 (44.7%)		16 (34.0%)		26 (55.3%)	
School	51 (46.8%)		60 (55.0%)		58 (53.2%)	
University	107 (65.6%)		96 (58.9%)		90 (55.2%)	
Ethnicity categorized
Pashtun	63 (50.4%)	0.465	61 (48.8%)	0.694	68 (54.4%)	0.313
Tajik	113 (57.4%)		101 (51.3%)		96 (48.7%)	
Others (Hazara, Uzbek, Turk)	53 (53.5%)		54 (54.5%)		57 (57.6%)	
Living with categorized
With parents, friends, or alone	117 (57.9%)	0.163	114 (56.4%)	0.043	104 (51.5%)	0.691
With wife and children	112 (51.1%)		102 (46.6%)		117 (53.4%)	
Economic status
High	70 (55.6%)	0.952	73 (57.9%)	0.153	66 (52.4%)	0.118
Medium	98 (53.8%)		85 (46.7%)		87 (47.8%)	
Low	61 (54.0%)		58 (51.3%)		66 (47.8%)	
Types of income
Constant	94 (59.1%)	0.129	84 (52.8%)	0.626	82 (59.0%)	0.086
Variable	135 (51.5%)		132 (50.4%)		73 (50.7%)	
Family members
≤5	79 (58.1%)	0.546	77 (56.6%)	0.055	53 (37.1%)	0.796
5–10	128 (52.2%)		125 (51.0%)		168 (60.4%)	
≥11	22 (55.0%)		14 (35.0%)		85 (49.4%)	
Living place
Urban	180 (53.1%)	0.277	184 (54.3%)	0.013	136 (54.6%)	0.814
Rural	49 (59.8%)		32 (39.0%)		47 (42.7%)	
BMI category
Underweight	21 (44.7%)	0.223	28 (59.6%)	0.426	92 (59.4%)	0.436
Normal weight	121 (53.5%)		116 (51.3%)		82 (52.6%)	
Overweight/Obese	87 (58.8%)		72 (48.6%)		69 (61.6%)	
Access to mass media?
Yes	221 (55.5%)	0.052	209 (52.5%)	0.039	102 (56.0%)	0.691
No	8 (34.8%)		7 (30.4%)		50 (39.4%)	
Have you heard about diabetes?
Yes	216 (55.0%)	0.381	204 (51.9%)	0.355	47 (46.1%)	0.003
No	13 (46.4%)		12 (42.9%)		26 (55.3%)	
Have you had an education about diabetes?
Yes	85 (75.9%)	<0.001	61 (54.5%)	0.435	58 (53.2%)	<0.001
No	144 (46.6%)		155 (50.2%)		90 (55.2%)	
Is there a family history of diabetes?
Yes	100 (68.5%)	<0.001	71 (48.6%)	0.423	68 (54.4%)	<0.001
No	129 (46.9%)		145 (52.7%)		96 (48.7%)	
If there is, then which type of diabetes?
Type 1	36 (58.1%)	0.014	34 (54.8%)	0.286	57 (57.6%)	0.056
Type 2	67 (77.0%)		40 (46.0%)		104 (51.5%)	
If you heard about diabetes, then what is your source?
Media	44 (53.7%)	0.001	45 (54.9%)	0.605	117 (53.4%)	0.028
HWCs	117 (64.3%)		95 (52.2%)		66 (52.4%)	
From friends, family, and others	68 (43.3%)		76 (48.4%)		87 (47.8%)	

In multivariable analysis for diabetes knowledge, participants aged 25–34 had significantly higher odds of good knowledge compared to those aged ≥35 (OR = 3.94, 95% CI: 1.04–14.99) (*p* = 0.044). School‐educated individuals had lower odds than university‐educated participants (OR = 0.19, 95% CI: 0.04–0.84) (*p* = 0.028), and those living in urban areas had significantly lower odds of good knowledge than rural residents (OR = 0.20, 95% CI: 0.05–0.87) (*p* = 0.032). Underweight participants had lower odds of good knowledge compared to overweight/obese individuals (OR = 0.11, 95% CI: 0.02–0.69) (*p* = 0.019), and having a family history of Type 1 diabetes was associated with lower knowledge than Type 2 (OR = 0.25, 95% CI: 0.09–0.69) (*p* = 0.007). For attitude, working 2–9 h per day significantly increased the odds of a good attitude compared to working 10 or more hours (OR = 3.41, 95% CI: 1.31–8.91) (*p* = 0.012), and participants with medium economic status had lower odds of good attitude than those with low income (OR = 0.23, 95% CI: 0.07–0.73) (*p* = 0.013). Regarding practice, males had significantly lower odds of good practice than females (OR = 0.13, 95% CI: 0.03–0.62) (*p* = 0.010), and single participants had lower odds compared to married individuals (OR = 0.20, 95% CI: 0.04–0.98) (*p* = 0.047). Those working 2–9 h daily had higher odds of good practice than those working ≥10 h (OR = 3.50, 95% CI: 1.10–11.07) (*p* = 0.033). Interestingly, participants who could read and write (without formal education) had significantly higher odds of good practice than university graduates (OR = 12.31, 95% CI: 1.74–87.10) (*p* = 0.012). Participants with high economic status had lower odds of good practice than those with low income (OR = 0.21, 95% CI: 0.05–0.93) (*p* = 0.040), and those who had received diabetes education had significantly greater odds of good practice (OR = 8.90, 95% CI: 2.67–29.63) (*p* < 0.001) (Table 6).

**TABLE 6:** Multivariable analysis of diabetes knowledge, attitude, and practice among community.

	High knowledge				High attitude				High practice			
			95% CI for OR				95% CI for OR				95% CI for OR	
Variables	Sig.	OR	Lower	Upper	Sig.	OR	Lower	Upper	Sig.	OR	Lower	Upper
Age groups
≥35 (ref.)												
18–24	0.518	1.733	0.328	9.164	0.353	0.519	0.130	2.074	0.640	0.681	0.136	3.399
25–34	0.044	3.940	1.036	14.994	0.322	0.576	0.193	1.717	0.483	1.570	0.445	5.545
Sex
Female (ref.)												
Male	0.385	0.510	0.112	2.331	0.244	2.262	0.574	8.918	0.010	0.134	0.029	0.616
Marital status
Currently married (ref.)												
Currently single	0.494	0.553	0.101	3.021	0.325	1.997	0.503	7.920	0.047	0.195	0.039	0.981
Occupations
Others (ref.)												
Employee/Unskilled worker	0.697	0.790	0.241	2.586	0.080	0.393	0.138	1.118	0.434	0.636	0.204	1.979
Housewife	0.810	1.199	0.274	5.245	0.196	0.423	0.115	1.557	0.411	0.544	0.127	2.323
Working hours per day
≥10 h (ref.)												
1–6 h	0.199	2.447	0.624	9.588	0.905	1.069	0.358	3.192	0.179	2.321	0.679	7.938
2–9 h	0.285	1.776	0.620	5.090	0.012	3.412	1.306	8.913	0.033	3.495	1.103	11.070
Education
University (ref.)												
Cannot read and write	0.833	0.835	0.157	4.442	0.188	0.382	0.091	1.600	0.293	2.435	0.463	12.812
Can read and write	0.909	0.898	0.142	5.678	0.328	0.453	0.093	2.214	0.012	12.308	1.739	87.104
School	0.028	0.191	0.044	0.838	0.728	1.249	0.357	4.371	0.440	1.712	0.437	6.708
Ethnicities
Others (Hazara, Uzbek, Turk)												
Pashtun	0.543	1.505	0.403	5.625	0.172	0.448	0.141	1.418	0.416	0.593	0.169	2.088
Tajik	0.052	3.520	0.987	12.554	0.303	0.574	0.200	1.651	0.588	0.717	0.216	2.386
Living with
With parents, friends, or alone (ref.)												
With wife and children	0.871	1.148	0.217	6.061	0.666	0.736	0.183	2.961	0.371	2.022	0.432	9.451
Economic status
Low (ref.)												
High	0.861	0.886	0.230	3.416	0.121	0.367	0.104	1.302	0.040	0.208	0.046	0.934
Medium	0.145	2.561	0.723	9.072	0.013	0.229	0.072	0.733	0.183	0.391	0.098	1.559
Type of income
Variable (ref.)												
Constant	0.254	0.562	0.209	1.513	0.871	1.073	0.460	2.500	0.264	1.757	0.653	4.724
Family members
≥11 (ref.)												
≤5	0.646	1.499	0.267	8.405	0.166	2.835	0.648	12.397	0.965	0.963	0.178	5.220
5–10	0.447	1.945	0.349	10.831	0.585	1.481	0.362	6.052	0.229	2.825	0.521	15.319
Residence
Rural (ref.)												
Urban	0.032	0.204	0.048	0.874	0.807	0.876	0.305	2.520	0.337	0.504	0.124	2.042
BMI
Overweight/Obese (ref.)												
Underweight	0.019	0.109	0.017	0.692	0.571	1.585	0.323	7.789	0.178	0.285	0.046	1.772
Normal	0.782	0.866	0.312	2.401	0.287	1.620	0.667	3.938	0.590	0.767	0.292	2.015
Mass media
No (ref.)												
Yes	0.286	4.812	0.269	86.048	0.195	4.221	0.479	37.233	0.055	14.038	0.947	208.024
Have you heard about diabetes?
No (ref.)												
Yes	0.556	2.058	0.186	22.775	0.839	0.800	0.093	6.903	0.370	2.992	0.272	32.865
Have you had an education about diabetes?
No (ref.)												
Yes	0.176	2.112	0.715	6.239	0.927	0.959	0.391	2.354	0.000	8.901	2.674	29.625
Is there family history of diabetes in your family?
No (ref.)												
Yes	0.121	0.069	0.002	2.033	0.444	0.319	0.017	5.946	0.997	1.006	0.063	16.076
If there is, then which type of diabetes?
Type 2 (ref.)												
Type 1	0.007	0.251	0.092	0.685	0.375	1.467	0.629	3.420	0.919	0.951	0.364	2.484
If you heard about diabetes, then what is your source?
From friends, family, and others (ref.)												
Media	0.999	0.786	0.430	2.466	0.973	0.965	0.127	7.318	0.507	0.490	0.060	4.034
HCWs	0.968	1.022	0.353	2.953	0.647	0.800	0.307	2.083	0.221	0.495	0.161	1.525
Constant	0.505	4.679			0.649	2.544			0.371	0.131		

## 4 Discussion

The primary goal of this study was to assess the KAP related to diabetes mellitus among hospital outpatients and their companions in Herat, Afghanistan. Our findings revealed that although the study population demonstrated a moderate understanding of diabetes—with 54.4% having good knowledge, 51.3% expressing positive attitudes, and 52.5% engaging in good practices—critical gaps remain, especially in dietary behaviors and blood glucose monitoring.

### 4.1 Knowledge

Our study found that 54.4% of participants demonstrated good knowledge about DM, whereas 45.6% had poor knowledge. This reflects a moderately informed population and aligns closely with findings from other developing settings. For example, in Malaysia, 54% of respondents had good knowledge, and in Ethiopia, 52.5% of people without diabetes were classified as knowledgeable [24, 25].

However, global comparisons reveal significant variation. In Sri Lanka, 77% of respondents demonstrated moderate to above‐moderate knowledge, and in Nepal, 60% of people without diabetes performed well on knowledge assessments [22, 26]. In contrast, only 25% of people without diabetes in **Saudi Arabia** showed good knowledge, and large knowledge gaps were also reported in **Bangladesh** [21, 27]. This variation may reflect differences in literacy, public health messaging, access to healthcare, and the extent of community exposure to chronic disease education across settings.

Sociodemographic analysis in our study revealed that females, university‐educated individuals, and those with a family history of diabetes had significantly higher knowledge levels. These trends were similarly observed in studies from Ethiopia and Bangladesh [21, 24]. Moreover, receiving information from healthcare providers was linked to higher knowledge scores, affirming findings from **Saudi Arabia** and **Malaysia** [25, 27]. These findings underscore the critical role of education, targeted communication, and family history in shaping public knowledge about diabetes [28, 29].

At the same time, the moderately good knowledge observed in Herat is notable given the limited structured diabetes education described in the local context. A likely explanation is that participants may have obtained information through **informal but meaningful channels**, such as healthcare encounters, advice from relatives, prior exposure to people living with diabetes within the family, and mass media. This interpretation is supported by the fact that prior diabetes education and family history were significantly associated with better knowledge in our sample. In Afghanistan, where family networks often play an important role in health‐related decisions, knowledge may spread socially even when formal diabetes education programs are limited.

### 4.2 Attitude

Most participants in Herat displayed positive attitudes toward diabetes, with 51.3% classified as having good attitudes. High endorsement was seen for routine diabetes screening (97.9%) and family support (88.6%). These results align with studies from **Nepal** (73.3%) and **Ethiopia** (55.9%), where favorable attitudes among people without diabetes were reported [24, 26]. Even in **Malaysia**, where only moderate knowledge was found, 61% of participants had a good attitude, suggesting that public health messaging can shape attitudes independent of deep understanding [25].

Demographically, single or previously married individuals, urban dwellers, the highly educated, and those with media access were significantly more likely to have favorable attitudes. These patterns mirror those reported in **Nepal**, **Ethiopia**, and **Bangladesh**, where mass media campaigns played a crucial role in improving attitudes [21, 24, 26]. In the context of Herat, these findings may indicate that attitudes are influenced not only by formal education but also by social exposure, family discussion, and access to television, radio, and internet‐based information. Even in the absence of a structured diabetes education system, repeated exposure to health messages through media and community experience may help shape positive perceptions about screening and prevention.

Still, Herat's performance in this domain is only modest when compared globally. Although better than **Sri Lanka**, where 90% demonstrated poor attitudes, Herat trails behind **Nepal** and other settings with more comprehensive diabetes education [22]. A significant proportion of participants remained neutral regarding the disease's impact on relationships and daily life, suggesting cultural hesitance, limited discussion of chronic illness within families, or insufficient awareness of the psychosocial burden of diabetes. In socially conservative settings such as Afghanistan, people may more readily acknowledge the medical importance of a disease than openly discuss its emotional, social, or marital consequences. This may help explain why attitudes toward screening were highly positive, whereas attitudes regarding the broader life impact of diabetes were less strong.

### 4.3 Practice

Encouragingly, 52.5% of respondents in Herat exhibited good diabetes‐related practices. This is comparable to Nepal (51.1%) and exceeds figures reported in Malaysia and Ethiopia, where poor practice was observed in 72% and 62.2% of people without diabetes, respectively [25, 30]. Behavioral highlights included high rates of medical consultation (80.8%) and physical activity (48.0%). These findings may suggest that many participants were at least somewhat engaged in health‐seeking behavior and self‐care, possibly because chronic illnesses such as diabetes are increasingly recognized within families and communities.

Notably, complete abstinence from alcohol and low tobacco use (79.8%) suggest that cultural and religious values may play a beneficial role in promoting some healthy behaviors. In Herat, where most of the population is Muslim, alcohol consumption is religiously prohibited and socially discouraged; therefore, the reported 0% alcohol use likely reflects this sociocultural context. At the same time, self‐reported alcohol use in interviewer‐administered surveys may also be influenced by social desirability bias, especially in conservative settings. These figures are more favorable than those from Nepal and Bangladesh [21, 26]. Nevertheless, concerning gaps remain in dietary control and glucose monitoring. A significant portion of participants reported frequent intake of fatty (37.5%) and sugary foods (34.0%), whereas 44.9% never checked their blood sugar—paralleling gaps noted in Bangladesh, Ethiopia, and Sri Lanka [21, 22, 30]. This pattern suggests that although general awareness and attitudes may be moderate, translating knowledge into sustained preventive behavior remains difficult. Possible explanations include limited access to regular screening, low perceived personal risk among people without diagnosed diabetes, financial barriers, and the influence of local dietary habits and family eating patterns.

Females, housewives, and those with fewer work hours demonstrated significantly better practices, mirroring behavioral patterns reported in Ethiopia and Nepal [24, 26]. In the Herat context, this may be because women and housewives are more involved in household food preparation and daily caregiving, which may increase their attention to diet and health‐related behaviors. Likewise, people working fewer hours may have more time to attend to self‐care or seek medical advice. Media was slightly more effective than healthcare providers in influencing practice, emphasizing the importance of wide‐reaching campaigns to improve community‐level behavior [31]. This may also suggest that television, radio, and online platforms are important sources of health information in Afghanistan and may offer practical opportunities for culturally appropriate diabetes education.

## 5 Recommendations

To improve diabetes prevention and management in Herat, targeted educational interventions should be integrated into primary care and delivered by trained professionals that have been shown to enhance KAP outcomes [32, 33]. Media and community‐based campaigns are essential, particularly targeting males, young adults, and low‐literacy populations. Outreach programs and structured follow‐up—especially in rural areas—can significantly enhance glycemic control and public awareness [34]. Promoting routine glucose monitoring, family engagement, and culturally appropriate health messaging will ensure long‐term improvement in diabetes‐related behaviors [35, 36, 37, 38].

## 6 Limitations

This study has several limitations. First, the sample was limited to hospital outpatients in urban Herat, which may not reflect the KAP levels of rural or non‐hospital populations. Second, the cross‐sectional design prevents establishing causal relationships between KAP. Third, reliance on self‐reported data introduces potential recall and social desirability bias, particularly in reporting health practices. Additionally, we did not assess participants’ actual glycemic control or biometric indicators, which could have validated reported behaviors. Finally, limited access to female participants in conservative areas may have affected the representativeness of gender‐based comparisons.

## 7 Conclusion

This study provides a comprehensive assessment of diabetes‐related KAP among hospital outpatients in Herat, Afghanistan. Although over half of the participants demonstrated good KAP levels, significant gaps remain—particularly in lifestyle practices such as dietary habits and blood glucose monitoring. Sociodemographic factors, including education level, gender, family history of diabetes, and access to health information, were significantly associated with KAP outcomes. The findings underscore the urgent need for targeted educational programs, culturally sensitive public health messaging, and community‐based interventions to address these gaps. Strengthening diabetes awareness through media, healthcare provider engagement, and structured follow‐up can play a vital role in promoting healthier behaviors and improving disease outcomes in resource‐limited settings like Afghanistan.

## Author Contributions

**Mohammad Masudi**: conceptualization, methodology, formal analysis, validation; writing – original draft, writing – review and editing, supervision. **Mohammad Shafi Saljuqi**: investigation, data curation, writing – review and editing. **Fatemah Rezaei**: investigation, data curation, writing – review and editing. **Zhila Arjmand**: investigation, data curation, writing – review and editing. **Parwin Arsin**: investigation, data curation, writing – review and editing. **Enayatollah Ejaz**: investigation, data curation, writing – review and editing. **Ali Rahimi**: conceptualization, methodology, formal analysis, writing – original draft, writing – review and editing. **Fahim Ahmadi**: investigation, data curation, writing – review and editing. **Nasar Ahmad Shayan**: methodology, formal analysis, writing – review and editing, supervision.

## Funding

The authors have nothing to report.

## Ethics Statement

This study was approved by the Institutional Review Board of Jami University (Approval No. 2024.1.27.6). Confidentiality was maintained, and data were anonymized at the point of collection. Participants were informed of their right to withdraw at any time without consequence. All procedures followed the ethical principles outlined in the Declaration of Helsinki.

## Consent

Consent was obtained from participants before heading to fill out the questionnaire. All participants provided written informed consent prior to participation.

## Conflicts of Interest

The authors declare no conflicts of interest.

## Data Availability

The datasets generated and/or analyzed during the current study are available from the corresponding author, Dr. Mohammad Masudi (mhmasoudy313@gmail.com), upon reasonable request.

## References

[1] World Health Organization , Diabetes, Geneva: World Health Organization; 2024, https://www.who.int/health-topics/diabetes#tab=tab_1.

[2] World Health Organization . Diabetes [fact sheet], Geneva: World Health Organization; 2024, https://www.who.int/news-room/fact-sheets/detail/diabetes.

[3] Y. Ezzatvar and A. García‐Hermoso , “Global Estimates of Diabetes‐Related Amputations Incidence in 2010–2020: A Systematic Review and Meta‐Analysis,” Diabetes Research and Clinical Practice 195 (2023): 110194, 10.1016/j.diabres.2022.110194. 36464091

[4] International Diabetes Federation , IDF Diabetes Atlas 2025 11th Edition. Brussels: International Diabetes Federation; 2025, https://diabetesatlas.org/resources/idf-diabetes-atlas-2025/.

[5] S. Shahraz , S. Saeedi Moghaddam , M. Azmin , et al., “Prevalence of Diabetes and Prediabetes, and Achievements in Diabetes Control in Iran; The Results of the STEPS of 2016,” Archives of Iranian Medicine 25 (2022): 591–599, 10.34172/aim.2022.94. 37543884 PMC10685770

[6] R. B. Rajput , Pakistan Has the World's Highest Diabetes Prevalence— and Lacks Focus on Prevention (Health Policy Watch, 2024), https://healthpolicy‐watch.news/pakistan‐has‐the‐worlds‐highest‐diabetes‐prevalence‐and‐lacks‐focus‐on‐prevention/.

[7] World Diabetes Foundation , Live longer now improving diabetes awareness and access in Afghanistan WDF15-1206. Bagsværd (Denmark): World Diabetes Foundation; 2016–2021, https://www.worlddiabetesfoundation.org/what‐we‐do/projects/wdf15‐1206/.

[8] Diabetes Mellitus in Afghanistan, World Life Expect (2025), https://www.worldlifeexpectancy.com/country‐health‐profile/afghanistan.

[9] A. D. Deshpande , M. Harris‐Hayes , and M. Schootman , “Epidemiology of Diabetes and Diabetes‐Related Complications,” Physical Therapy 88 (2008): 1254–1264, 10.2522/ptj.20080020. 18801858 PMC3870323

[10] T. R. Einarson , A. Acs , C. Ludwig , and U. H. Panton , “Prevalence of Cardiovascular Disease in Type 2 Diabetes: A Systematic Literature Review of Scientific Evidence From Across the World in 2007–2017,” Cardiovascular Diabetology 17 (2018): 83, 10.1186/s12933-018-0728-6. 29884191 PMC5994068

[11] American Diabetes Association ; “Diabetic Retinopathy,” Diabetes Care January 25 suppl_1 (2002): s90–s93, 10.2337/diacare.25.2007.S90.

[12] D. T. Nguyen and E. A. Graviss , “Diabetic Trends and Associated Mortality in Tuberculosis Patients in Texas, a Large Population‐Based Analysis,” Tuberculosis 116 (2019): S59–65, 10.1016/j.tube.2019.04.011. 31064712

[13] M. Koopmanschap , “Coping With Type II Diabetes: The Patient's Perspective,” Diabetologia 45 (2002): S21–S22, 10.1007/s00125-002-0861-2. 27942780

[14] W. Mansy , S. Wajid , A. Alwhaibi , et al., “Assessing Outpatients′ Knowledge, Attitude, and Practice Toward Managing Diabetes in Saudi Arabia,” Inquiry: The Journal of Health Care Organization, Provision, and Financing 59 (2022): 00469580221082781, 10.1177/00469580221082781. PMC8984850 35377247

[15] N. K. Srinivasan , D. John , G. Rebekah , E. S. Kujur , P. Paul , and S. S. John , “Diabetes and Diabetic Retinopathy: Knowledge, Attitude, Practice (KAP) Among Diabetic Patients in a Tertiary Eye Care Centre,” Journal of Clinical and Diagnostic Research (JCDR) 11 (2017): NC01–NC07, 10.7860/JCDR/2017/27027.10174. PMC5583928 28892947

[16] J. Jin , G. E. Sklar , V. Min Sen Oh , and S. Chuen Li , “Factors Affecting Therapeutic Compliance: A Review From the Patient's Perspective,” Therapeutics and Clinical Risk Management 4 (2008): 269–286, 10.2147/tcrm.s1458. 18728716 PMC2503662

[17] L. M. Otero , M. L. Zanetti , and M. D. Ogrizio , “Knowledge of Diabetic Patients About Their Disease Before and After Implementing a Diabetes Education Program,” Revista Latino‐Americana De Enfermagem 16 (2008): 231–237, 10.1590/S0104-11692008000200010. 18506341

[18] D. Mohan , D. Raj , C. S. Shanthirani , et al., “Awareness and Knowledge of Diabetes in Chennai—The Chennai Urban Rural Epidemiology Study [CURES‐9],” Journal of the Association of Physicians of India 53 (2005): 283–287. 15987011

[19] K. M. Islam Saeed , “Diabetes Mellitus Among Adults in Herat, Afghanistan: A Cross‐Sectional Study,” Central Asian Journal of Global Health 6 (2017): 271, 10.5195/cajgh.2017.271. 29138737 PMC5675391

[20] S. H. Fayaz , N. Hamajima , M. K. Frozanfar , et al., “Factors Associated With Diabetes Mellitus and Hypertension Among Adults in the Northern Rural Area, Afghanistan,” Nagoya Journal of Medical Science 86 (2024): 564–577, 10.18999/nagjms.86.4.564. 39780927 PMC11704768

[21] K. Fatema , S. Hossain , K. Natasha , et al., “Knowledge Attitude and Practice Regarding Diabetes Mellitus Among Nondiabetic and Diabetic Study Participants in Bangladesh,” BMC Public Health [Electronic Resource] 17 (2017): 364, 10.1186/s12889-017-4285-9. 28446194 PMC5406895

[22] H. M. M. Herath , N. P. Weerasinghe , H. Dias , and T. P. Weerarathna , “Knowledge, Attitude and Practice Related to Diabetes Mellitus Among the General Public in Galle District in Southern Sri Lanka: A Pilot Study,” BMC Public Health [Electronic Resource] 17 (2017): 535, 10.1186/s12889-017-4459-5. 28571566 PMC5455097

[23] M. Alsous , M. A. Jalil , M. Odeh , R. A. Kurdi , and M. Alnan , “Public Knowledge, Attitudes and Practices Toward Diabetes Mellitus: A Cross‐Sectional Study From Jordan,” PLoS ONE 14 (2019): e0214479, 10.1371/journal.pone.0214479. 30925187 PMC6440628

[24] C. W. Kassahun and A. G. Mekonen , “Knowledge, Attitude, Practices and Their Associated Factors Towards Diabetes Mellitus Among Non Diabetes Community Members of Bale Zone Administrative Towns, South East Ethiopia. A Cross‐Sectional Study,” PLoS ONE 12 (2017): e0170040, 10.1371/journal.pone.0170040. 28152066 PMC5289457

[25] A. Binti Mohd Baki , A Cross‐Sectional Study on the Level of Knowledge, Attitude and Practice on Diabetes Mellitus Among Population Aged 18 Years Old and Above in Rumah Baseh, Bawang Assan, Sibu From 25th May Until 7th August 2009 (Universiti Malaysia Sarawak, 2009).

[26] Y. Sapkota , “Knowledge, Attitude and Practice of Type 2 Diabetic Patients Visiting Diabetic OPD of TUTH and Non Diabetic Population of Kathmand,” Journal of Diabetes and Endocrinology Association of Nepal 2 (2018): 17–23, 10.3126/jdean.v2i1.21195.

[27] S. M. B. Asdaq , “Knowledge, Attitude, and Practice Regarding Diabetes Mellitus Among General Public and Diabetic Patients in Riyadh, Saudi Arabia,” Asian Journal of Pharmaceutics (AJP) 12 (2018): 268–276, 10.22377/ajp.v12i01.2071.

[28] R. Dobson , K. Carter , R. Cutfield , et al., “Diabetes Text‐Message Self‐Management Support Program (SMS4BG): A Pilot Study,” JMIR mHealth and uHealth 3 (2015): e3988, 10.2196/mhealth.3988. PMC4390615 25830952

[29] A. Seth , “Challenges of Achieving an Optimum Glycemic Control in Children With Type 1 Diabetes in India,” Indian Journal of Pediatrics 87 (2020): 491–492, 10.1007/s12098-020-03336-6. 32410002

[30] A. Simachew and H. Temesgen , “Knowledge, Attitude, Practice, and Their Associated Factor Towards Diabetes Mellitus Among Peoples Live in Debre Markos Town, North West Ethiopia, Amhara Regional State, Ethiopia 2020 GC,” preprint, Research Square, January 5, 2022, 10.21203/rs.3.rs-1138353/v1.

[31] S. H. Kim , Y. Kim , S. Choi , and B. Jeon , “Evaluation of Nurse‐Led Social Media Intervention for Diabetes Self‐Management: A Mixed‐Method Study,” Journal of Nursing Scholarship 54 (2022): 569–577, 10.1111/jnu.12770. 35174636

[32] M. M. Alsous , M. Odeh , and M. Abdel Jalil , “Effect of an Educational Intervention on Public Knowledge, Attitudes, and Intended Practices Towards Diabetes Mellitus: A Quasi‐Experimental Study,” International Journal of Clinical Practice 74 (2020): e13565, 10.1111/ijcp.13565. 32474991

[33] S. Nkomani , S. Ruskaniko , and R. Blaauw , “The Impact of Existing Diabetes Self‐Management Education Interventions on Knowledge, Attitudes and Practices in Public Health Care Institutions in Harare, Zimbabwe,” South African Journal of Clinical Nutrition 34 (2021): 27–33, 10.1080/16070658.2019.1641272.

[34] L. Qi , L. Feng , W. Tang , et al., “A Community‐Based Comprehensive Intervention Program for 7200 Patients With Type 2 Diabetes Mellitus in Chongqing (China),” International Journal of Environmental Research and Public Health 11 (2014): 11450–11463, 10.3390/ijerph111111450. 25383608 PMC4245623

[35] R. A. Ajjan , T. Battelino , X. Cos , et al., “Continuous Glucose Monitoring for the Routine Care of Type 2 Diabetes Mellitus,” Nature Reviews Endocrinology 20 (2024): 426–440, 10.1038/s41574-024-00973-1. 38589493

[36] N. Ehrhardt , B. Cedeno , L. Montour , et al., “Effectiveness of a Culturally Tailored Diabetes Education Curriculum With Real‐Time Continuous Glucose Monitoring in a Latinx Population With Type 2 Diabetes: The CUT‐DM With CGM for Latinx Randomised Controlled Trial Study Protocol,” BMJ Open 13 (2023): e082005, 10.1136/bmjopen-2023-082005. PMC10759074 38154895

[37] C. Higa , E. J. Davidson , and J. R. Loos , “Integrating Family and Friend Support, Information Technology, and Diabetes Education in Community‐Centric Diabetes Self‐Management,” Journal of the American Medical Informatics Association 28 (2021): 261–275, 10.1093/jamia/ocaa223. 33164074 PMC7883992

[38] P. A. McElfish , S. Riklon , R. S. Purvis , et al., “Study Protocol for Family Model Diabetes Self‐Management Education With Marshallese Participants in Faith‐Based Organizations,” Contemporary Clinical Trials Communications 30 (2022): 101007, 10.1016/j.conctc.2022.101007. 36186543 PMC9515595

